# Construction of a novel mRNA-signature prediction model for prognosis of bladder cancer based on a statistical analysis

**DOI:** 10.1186/s12885-021-08611-z

**Published:** 2021-07-27

**Authors:** Jianpeng Li, Jinlong Cao, Pan Li, Zhiqiang Yao, Ran Deng, Lijun Ying, Junqiang Tian

**Affiliations:** 1 Department of Urology, The Second Hospital of Lanzhou University, Lanzhou, China; 2 Key Laboratory of Gansu Province for Urological Diseases, Lanzhou, China; 3 Clinical Center of Gansu Province for Nephron-urology, Lanzhou, China

**Keywords:** Bladder cancer (BC), Differentially expressed genes (DEGs), Overall survival (OS), Risk score, Nomogram, TCGA, GEO

## Abstract

**Background:**

Bladder cancer (BC) is a common malignancy neoplasm diagnosed in advanced stages in most cases. It is crucial to screen ideal biomarkers and construct a more accurate prognostic model than conventional clinical parameters. The aim of this research was to develop and validate an mRNA-based signature for predicting the prognosis of patients with bladder cancer.

**Methods:**

The RNA-seq data was downloaded from the Cancer Genome Atlas (TCGA) and Gene Expression Omnibus (GEO). Differentially expressed genes (DEGs) were screened in three datasets, and prognostic genes were identified from the training set of TCGA dataset. The common genes between DEGs and prognostic genes were narrowed down to six genes via Least Absolute Shrinkage and Selection Operator (LASSO) regression, and stepwise multivariate Cox regression. Then the gene-based risk score was calculated via Cox coefficient. Time-dependent receiver operating characteristic (ROC) and Kaplan-Meier (KM) survival analysis were used to assess the prognostic power of risk score. Multivariate Cox regression analysis was applied to construct a nomogram. Decision curve analysis (DCA), calibration curves, and time-dependent ROC were performed to assess the nomogram. Finally, functional enrichment of candidate genes was conducted to explore the potential biological pathways of candidate genes.

**Results:**

*SORBS2, GPC2, SETBP1, FGF11, APOL1*, and *H1–2* were screened to be correlated with the prognosis of BC patients. A nomogram was constructed based on the risk score, pathological stage, and age. Then, the calibration plots for the 1-, 3-, 5-year OS were predicted well in entire TCGA-BLCA patients. Decision curve analysis (DCA) indicated that the clinical value of the nomogram was higher than the stage model and TNM model in predicting overall survival analysis. The time-dependent ROC curves indicated that the nomogram had higher predictive accuracy than the stage model and risk score model. The AUC of nomogram time-dependent ROC was 0.763, 0.805, and 0.806 for 1-year, 3-year, and 5-year, respectively. Functional enrichment analysis of candidate genes suggested several pathways and mechanisms related to cancer.

**Conclusions:**

In this research, we developed an mRNA-based signature that incorporated clinical prognostic parameters to predict BC patient prognosis well, which may provide a novel prognosis assessment tool for clinical practice and explore several potential novel biomarkers related to the prognosis of patients with BC.

**Supplementary Information:**

The online version contains supplementary material available at 10.1186/s12885-021-08611-z.

## Background

Bladder cancer (BC) is the tenth most commonly diagnosed carcinoma, with an estimated 549,000 new cases and 200,000 deaths reported globally in 2018, and BC ranks the first in urinary malignant neoplasm among males [1]. Therefore, it is crucial to developed accurate prognostic tools for predicting clinical results to help clinicians make decisions about treatment, drug therapy, and conservation options [2].

Conventional signatures used to predict overall survival (OS) can range from tumor clinical parameters and tumor pathology to special mutated genes. For instance, the tumor node metastasis (TNM) classification system is the most frequently utilized to predict the prognosis of cancer patients [3, 4]. Zhang et al. constructed a prediction tool based on clinical parameters to predict the survival of patients with BC [5]. The most significant advantage of TNM is straightforward, but the inevitable disadvantage is not an individualized prediction for each patient [6]. Besides, an increasing number of single signatures have been explored to predict the OS of BC patients, such as *OIP5* [7], *B4GALT1* [8], *ASPM* [9], and *HMGA2* [10]. Xie et al. utilized the expression of *B4GALT1* to predict the prognosis of patients with muscle-invasive bladder cancer, and the expression of *B4GALT1* was correlated with OS of patients with BC [8]. However, it is a challenge to predict the OS of patients with BC using a single signature, because of the impact of genetic heterogeneity [11]. Therefore, it is essential to develop a comprehensive prognostic evaluation system that can improve the predictive accuracy of the prognosis of patients with BC.

Nowadays, gene-based prognostic signatures in conjunction with other clinical parameters have been explored extensively in predicting the OS of cancer patients [12–14]. Song et al. identified signature combined immune-related genes and clinical characters to predict the OS of patients with BC, which suggested the signature was clinically useful for patients with BC [15]. And a growing number of studies have shown that prognostic signatures dependent on gene expression levels have a strong potential to predict the prognosis of cancer patients [16]. Therefore, in-depth analysis of gene expression databases may discover other prognostic genes and establish a robust prognostic signature, which can be a powerful tool for predicting cancer prognosis and individualized care [13].

In our study, we developed a signature to predict OS of BC patients based on multiple prognostic genes and clinical parameters. The RNA-seq was downloaded from TCGA and GEO, and analyzed via DEGs analysis. Then we utilized univariate Cox regression, LASSO regression with tenfold cross-validation, and stepwise multivariate Cox regression to identify six candidate genes. And the gene-based risk score was calculated through the stepwise multivariate cox coefficient multiplied by the expression of the gene. Then a nomogram was constructed based on the risk score and clinical parameters, which was assessed by the calibration plot, decision curve analysis (DCA), and time-dependent ROC analysis. Finally, potential pathways of these candidate genes were analyzed via functional enrichment analysis, Gene Ontology (GO) enrichment, and Kyoto Encyclopedia of Genes and Genomes (KEGG). Bioinformatic methods “guilt by association” (GBA) [17] and Gene set enrichment analysis (GSEA) were applied to explore the mechanism of candidate genes.

## Materials and methods

### Data source

Our study applied public datasets to conduct analysis based on the Cancer Genome Atlas (TCGA, https://portal.gdc.cancer.gov/) and Gene Expression Omnibus (GEO, https://www.ncbi.nlm.nih.gov/geo/). Gene expression data and corresponding clinical data were obtained from TCGA website via “gdc-client” tool. GEO datasets included GSE13507 [18] and GSE133624 [19].

### Differential expression genes analysis

The gene expression data of GSE13507 was conducted the DEGs analysis via R package “limma” [20]. For gene expression data of GSE133624 and TCGA-BLCA, the DEGs analysis was conducted via R package “DESeq2” [21]. After these analyses, the downregulated or upregulated gene was defined with adjust *P* value < 0.05, the |log2 fold change| > 1 [12, 22]. The shared DEGs among three datasets were showed in Venn diagram by R package “VennDiagram” [23].

### Selection of prognostic genes and validation of prognostic genes

In this part, we excluded the samples without corresponding survival data and clinical data. Then we divided bladder cancer data of TCGA into training set and testing set randomly. The information about training set and testing set is shown in Table 1. The univariate Cox analysis was utilized to select prognostic genes via R package “survival” in training set [24], which were obtained with the threshold of *P* < 0.05. The overlapping candidate genes (OCGs) were obtained by intersection analysis between prognostic genes and shared DEGs.

**Table 1:** The clinical information on training set and testing set

Type		Training (243)	Testing (162)	χ^2^	p
Gender	female	68	37	1.34	0.247
	male	175	125		
Race	American	12	11	10.7	0.014
	White	206	117		
	Asian	17	26		
	Not reported	8	8		
Pathologic-M	M0	108	86	3.35	0.187
	M1	8	3		
	MX	126	72		
Pathologic-N	N0	134	101	4.81	0.307
	N1	27	19		
	N2	53	22		
	N3	4	4		
	NX	22	14		
Stage	StageI	0	2	5.38	0.146
	StageII	78	51		
	StageIII	77	61		
	StageIV	87	47		

### Establishment of multiple-gene prognostic signature

We utilized LASSO regression with tenfold cross-validation to narrow down OCGs by R package “glmnet” [25]. A gene-based prognostic signature was constructed via stepwise multivariate Cox regression. Risk score based on gene prognostic signature was calculated for each TCGA-BLCA patient via gene expression multiplied by the regression coefficient in stepwise multivariate Cox regression.

### Estimation and validation of the multi-gene model

The testing set (*n* = 162) and the whole set (*n* = 405) were utilized to assess the predictive validity of the multi-gene prognostic signature. In the validation set, the risk score of each patient was calculated via the coefficient of the candidate genes obtained above. Then the patients were stratified into high-risk and low-risk groups based on the median risk score as the cutoff. The Kaplan-Meier (KM) survival analysis with log-rank test and time-dependent receiver operating characteristic (ROC) analysis was applied to validate the gene-based prognostic signature. Furthermore, the mutation type of selected genes was explored in cBioPortal (https://www.cbioportal.org/) [26, 27].

### Construction and validation of the prognostic nomogram

Based on risk score and some clinical parameters, a nomogram was established to predict the probability of 1-year, 3-year, and 5-year OS using R package “rms” [28]. The score of the prediction of nomograms for each patient was calculated via R package “nomogramFormular” [29]. With the source code provided on the MSKCC website(https://www.mskcc.org/departments/epidemiology-biostatistics/biostatistics/decision-curve-analysis), we performed the DCA analysis of survival outcome [6]. The calibration curve analysis was conducted via “calibrate” function of “rms” R package [28]. The time-dependent ROC analysis for nomogram score was performed via R package “timeROC” [30].

### Functional analysis and correlation analysis of genes in model

Gene ontology (GO) enrichment and Kyoto Encyclopedia of Genes and Genomes (KEGG) pathways analysis of candidate genes were performed by R package “clusterProfiler” [31, 32]. The threshold for analysis was set *P*-value < 0.05, indicating significantly enriched functional annotations. Bioinformatic methods “guilt by association” (GBA) [17] and GSEA were applied to conduct potential functional analysis. GSEA was conducted in R with R package “clusterProfiler” [32]. GBA was performed with Spearman method.

### Statistical analysis

The samples in TCGA were randomly divided into training set and testing set with “sample” function of R. Heatmap of DEGs obtained in three datasets were plotted with R package “pheatmap” [33]. Two groups of boxplots were analyzed with Wilcoxon-test. The comparison of clinical parameters between training set and testing set was conducted with **χ**^**2**^ test or exact Fisher test. As for KM survival analysis, *P*-value and hazard ratio (HR) was generated via log-rank tests and univariate Cox proportional hazards regression. All analysis above and R packages were performed in R software version 3.6.3 (The R Foundation for Statistical Computing, 2020). All statistical tests were two-sided. *P* < 0.05 was regarded as statically.

## Results

### Identification of DEGs

**Fig. 1:**
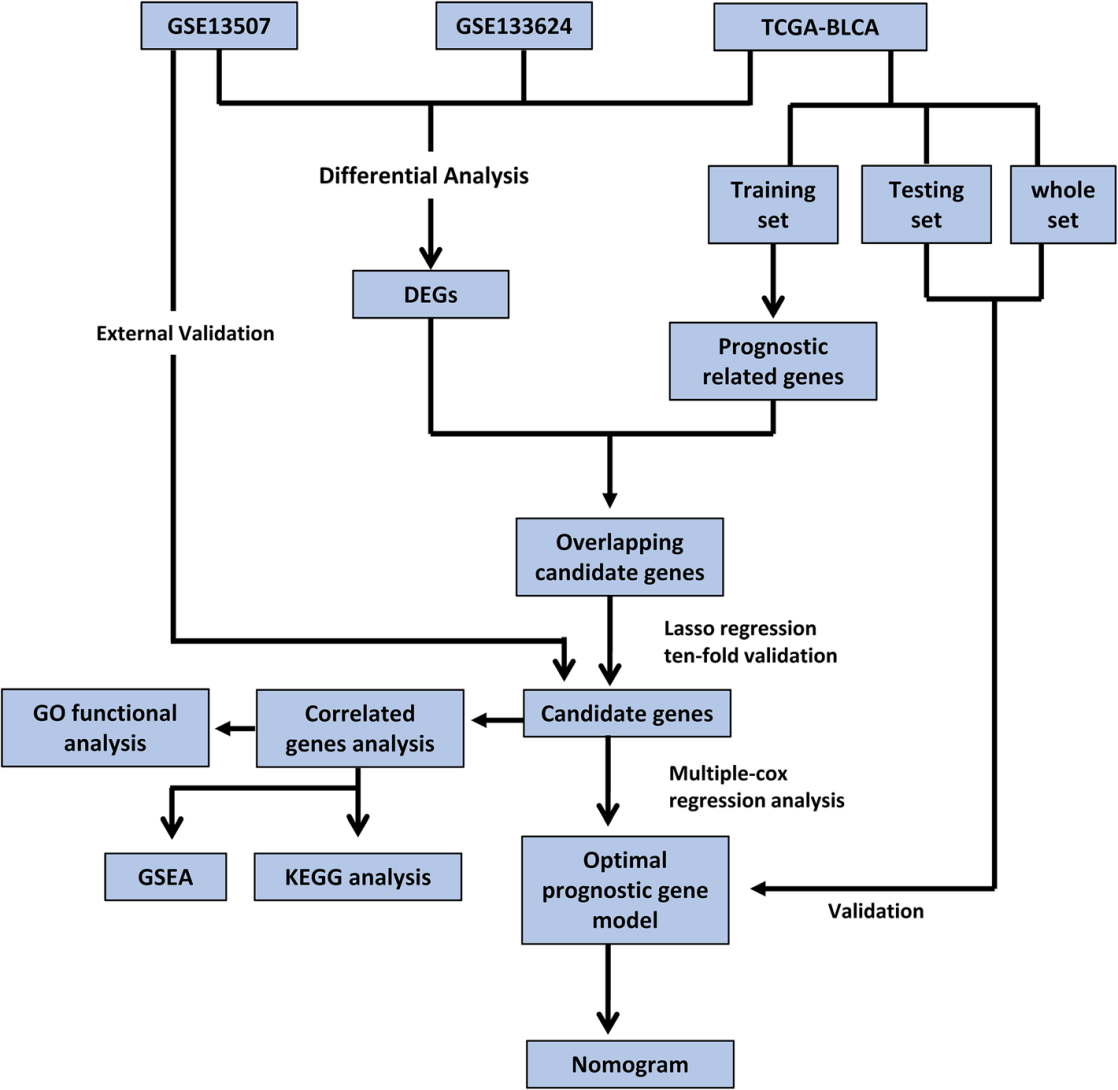
Flowchart of the whole study

**Fig. 2:**
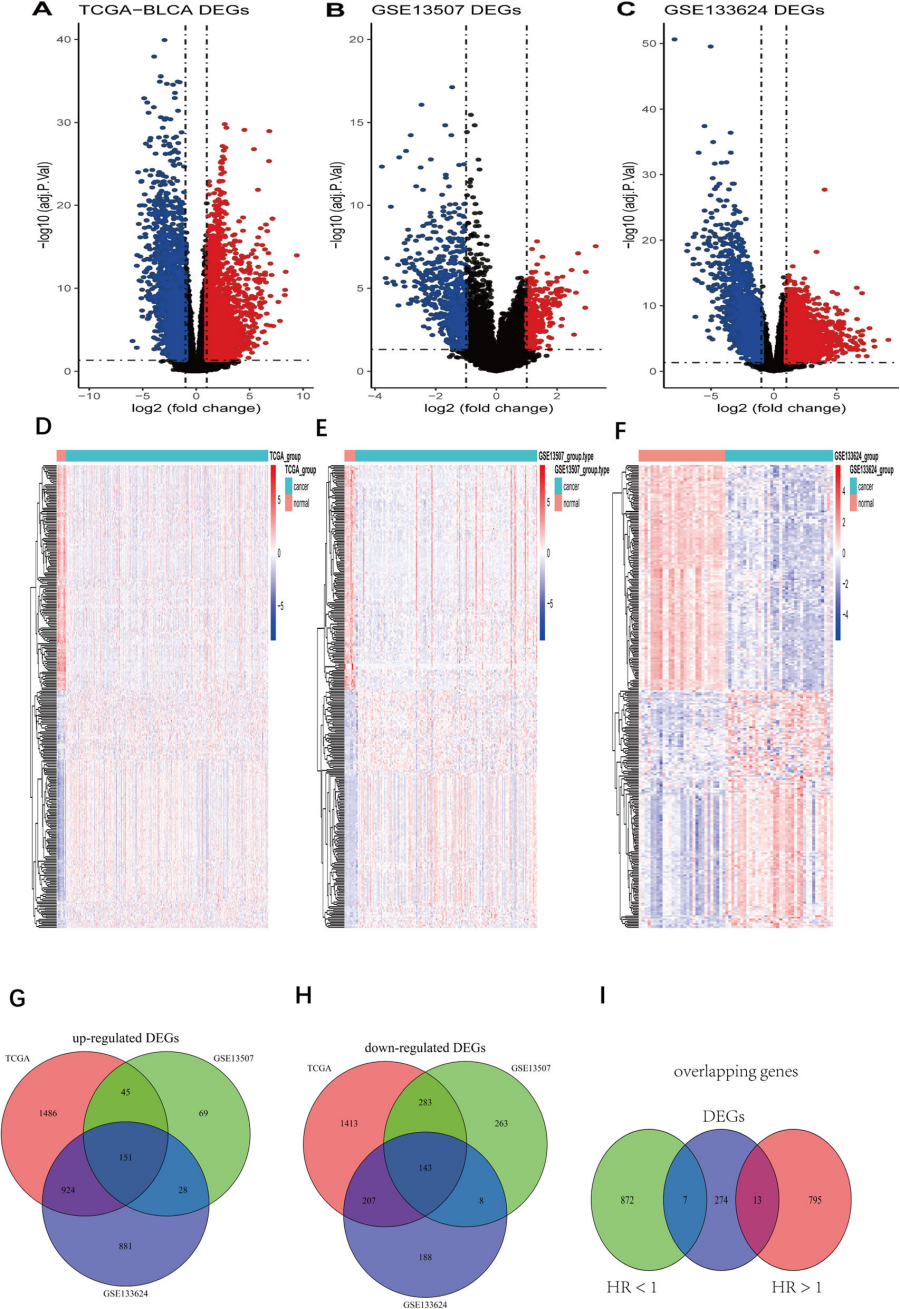
Differential gene expression analysis. **A-C:** Volcano plot of differentially expressed genes in BC tissue when compared with normal tissue in 3 datasets. Red nodes represent the significantly up-regulated genes with logFC > 1 and *p* < 0.05. Blue nodes represent the significantly down-regulated genes with logFC < − 1 and p < 0.05. **D-F:** Heatmap of common DEGs obtained in 3 datasets in BC. **G:** Venn diagram for up-regulated genes in 3 datasets. **H:** Venn diagram for down-regulated genes in 3 datasets. **I:** Venn diagram for DEGs and prognosis-related genes. DEGs: Differentially expressed genes. logFC**:** Log2-based fold change

The flowchart of this study is shown in Fig. 1. According to the differential gene selection criteria for differential analysis, 2606 up-regulated genes and 2046 down-regulated differential genes were screened in the TCGA-BLCA (Fig. 2 A, D). 293 up-regulated genes and 697 down-regulated genes were screened in GSE13507 (Fig. 2 B, E). 1984 up-regulated genes and 546 down-regulated genes were screened in GSE133624 (Fig. 2 C, F). Taking the intersection of the up-regulated and down-regulated genes in the three data sets, 151 up-regulated genes and 143 down-regulated genes were obtained (Fig. 2 G, H).

### Selection of prognostic genes

Based on the univariate Cox analysis, 808 prognosis-related genes (HR > 1) and 879 prognosis-related genes (HR < 1) were screened in training set. Then the prognostic genes were intersected with 294 DEGs. Finally, 20 shared genes were obtained, which includes 7 DEGs with HR < 1 and 13 DEGs with HR > 1 (Fig. 2 I).

### Establishment of six-gene-based model

**Fig. 3:**
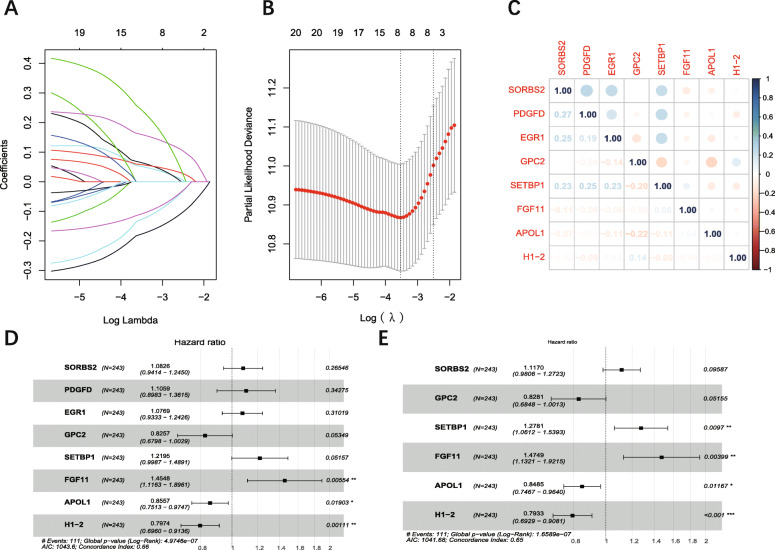
Identification of 6 prognostic genes in BC. **A:** LASSO coefficients profiles of protein-coding genes. **B:** LASSO-penalized regression with tenfold cross-validation obtained 8 genes using minimum lambda value. **C:** correlation score of eight genes obtained from LASSO-penalized regression. **D:** Multivariate Cox regression analysis of eight genes. **E:** Multivariate Cox regression analysis of eight genes with stepwise regression. ***:*P* < 0.001; **: *P* < 0.01: *: *P* < 0.05. LASSO: Least Absolute Shrinkage and Selection Operator

LASSO regression with tenfold cross-validation was conducted to get the optimal lambda value from the minimum partial likelihood deviance (λ_min_ = 0.03522) (Fig. 3 A, B) [12]. Prognostic DEGs were narrowed down to an eight-gene signature. The correlation analysis showed that the expression of these eight genes was not significant (Fig. 3 C), which means the signature based on these genes was not overfitting. Six candidate genes (*SORBS2, GPC2, SETBP1, FGF11, APOL1, H1–2*) were selected via stepwise multivariate Cox regression (Fig. 3 D, E). Then six-gene-based signature (Risk score = 0.11061*Exp _(SORBS2)_ - 0.18866*Exp _(GPC2)_ + 0.24538*Exp _(SETBP1)_ + 0.38858*Exp _(FGF11)_ – 0.16433*Exp _(APOL1)_ -0.23161*Exp _(H1–2)_) was constructed.

**Fig. 4:**
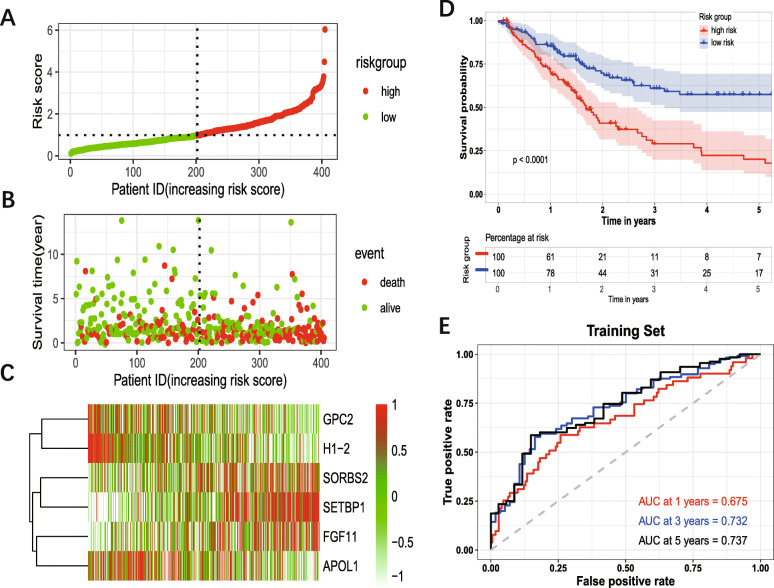
Prognostic analysis of six-gene signature. The dotted line represented the median risk score and divided the patients into low- and high-risk group. **A:** The curve of risk score. **B:** Survival status of the patients. More dead patients corresponding to the higher risk score. **C:** Heatmap of the expression profiles of the six prognostic genes in low- and high-risk group. **D:** Kaplan–Meier survival analysis of the six-gene signature. **E:** Time-dependent ROC analysis of the six-gene signature. ROC: receiver operating characteristic

The patients were divided into the high-risk group and low-risk group based on the median risk score as cutoff (Fig. 4 A). Figure 4 B showed the survival status of the patients. And Fig. 4 C showed the heatmap of six prognosis-related genes. The KM survival analysis for the training set showed that the high-risk group had a worse OS compared with the low-risk group (Fig. 4 D). The AUC of 1-year, 3-year, 5-year for training set were 0.635, 0.732, 0.737, respectively (Fig. 4 E).

### Expression analysis, mutation analysis, and protein level analysis of genes in the prognostic model

**Fig. 5:**
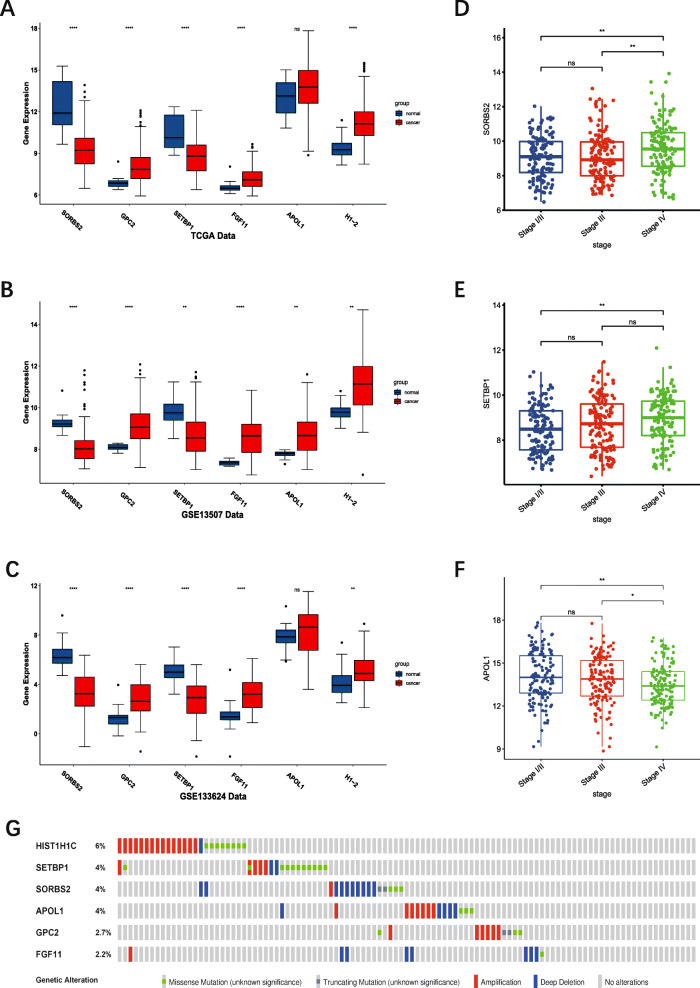
Expression and mutation of prognostic genes. **A:** Expression pattern of the six prognostic genes between tumor and normal bladder tissue. **B-D:** Expression of *SORBS2, SETBP1, APOL1,* among different pathological stages. **E:** A visual summary across on a query of 6 prognostic genes showing genetic alteration of these six genes in TCGA-BLCA patients. ***:*P* < 0.001, **: *P* < 0.01, *: *P* < 0.05, ns: not significant

**Fig. 6:**
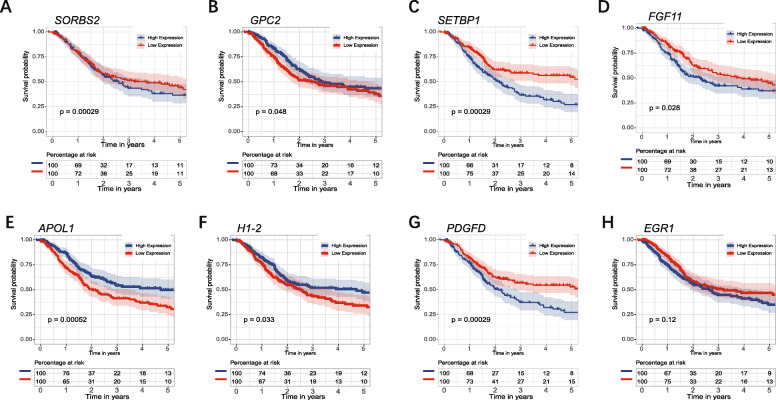
Kaplan–Meier survival analysis of *SORBS2, GPC2, SETBP1, FGF11, APOL1,* and *H1–2*. The expression of *SORBS2, GPC2, SETBP1, FGF11, APOL1, H1–2*, and *PDGFD* were significantly correlated with OS of patients with BC

The expression of six genes in tumor and normal tissue were shown in Fig. 5 A-C, which indicated that *GPC2, FGF11, APOL1*, and *H1–2* were highly expressed in tumor tissue, while *SORBS2* and *SETBP1* were highly expressed in normal tissue. The expression of six genes in TCGA-BLCA database indicated that *SORBS2, SETBP1, APOL1* were differently expressed in different stages. *SORBS2* and *SETBP1* were significantly up-regulated in stage IV (Fig. 5 D, E). *APOL1* was significantly up-regulated in stage I/II (Fig. 5 F). Therefore, these three genes may be associated with the pathological stage of BC. The genetic alteration type of six genes was analyzed in the cBioPortal database (Fig. 5 G). KM survival analysis for each prognostic gene showed that the expression of *GPC2, SETBP1, FGF11, APOL1, H1–2, PDGFD* were significantly correlated with OS of patients with BC (Fig. 6). Moreover, the immunohistochemistry from Human Protein Atlas database (Supplementary Fig. S1) showed that *APOL1, GPC2*, and *H1–2* had a higher protein level in urothelial cancer, while *SETBP1* had a higher protein level in the urinary bladder. This result was consistent with the mRNA analysis in TCGA database.

### Validation of the six-gene prognostic signature

**Fig. 7:**
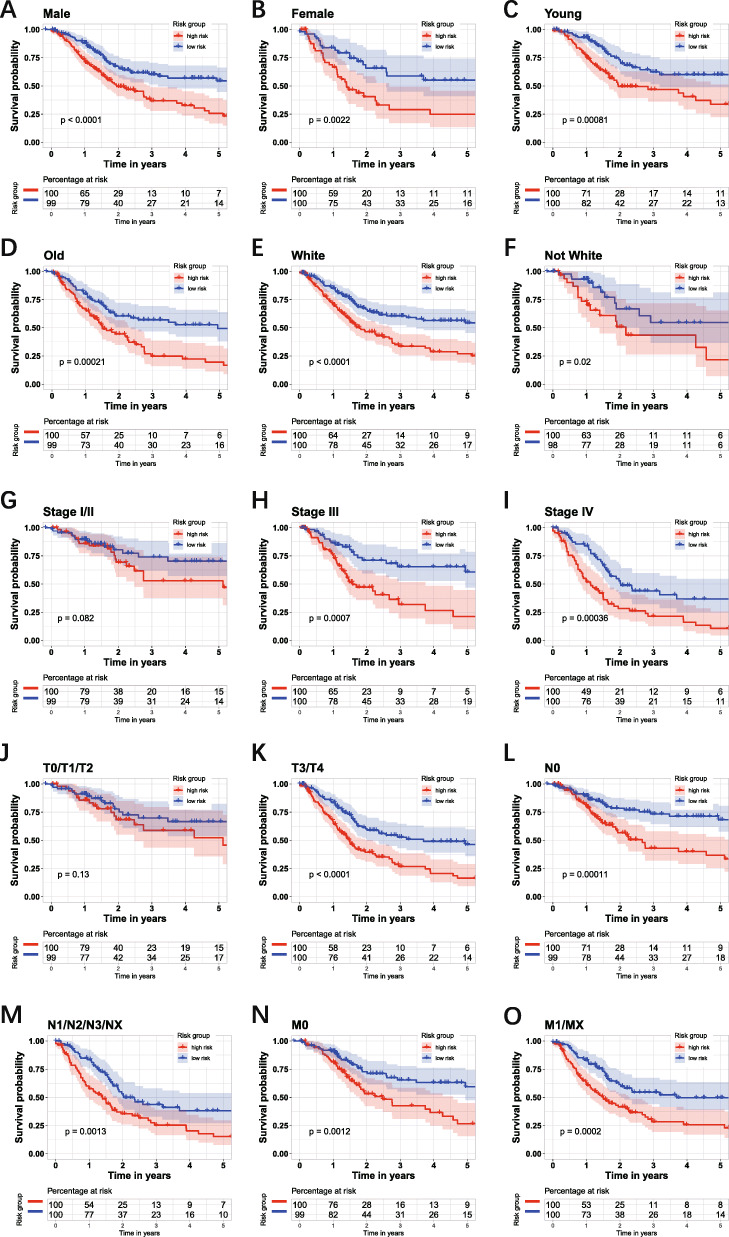
Kaplan–Meier survival analysis of the six-gene risk score level in different subgroups. The group of age (**A-B**), gender (**C-D**), race (**E-F**), AJCC-N (**L-M**), AJCC-M (**N-O**) indicated that the patients with high-risk score had significantly worse OS. In the group of AJCC-stage, the patients with the high-risk score in the stage I/II did not have significantly worse OS (**G**), while in stage III and stage IV, the patients with the high-risk score have significantly worse OS (**H-I**). In the group of AJCC-T, the patients with high-risk score in T0/1/2 did not have significantly worse OS (**J**), while in T3/4 group, the patients with the high risk-score have worse OS (**K**)

**Fig. 8:**
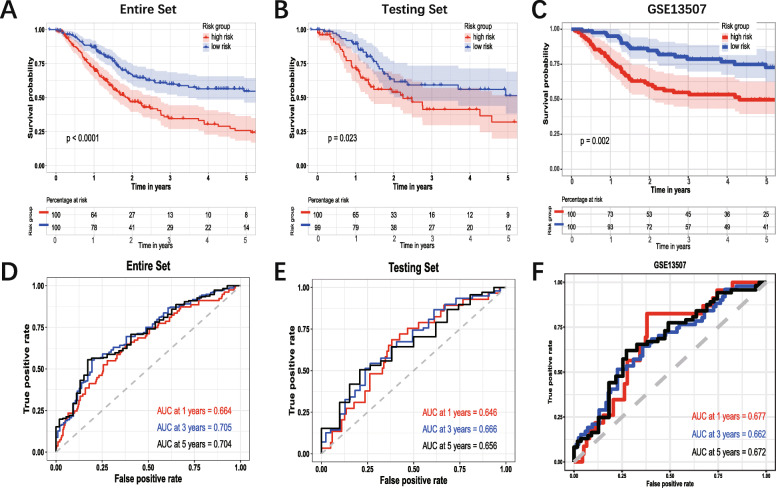
Validation of the six-gene signature. Kaplan–Meier survival analysis of the 6-gene signature in validation set. **A:** The whole set. **B:** The testing set. **C:** The external dataset GSE13507. **D:** Time-dependent ROC analysis of the six-gene signature in whole set. **E:** Time-dependent ROC analysis of the six-gene signature in testing set. F: Time-dependent ROC analysis of the six-gene signature in GSE13507 dataset

The survival analysis in different subgroups showed that the risk score had a satisfactory performance. The group of age (Fig. 7A-B), gender (Fig. 7C-D), race (Fig. 7E-F), AJCC-N (Fig. 7 L-M), AJCC-M (Fig. 7N-O) indicated that the patients with high-risk score had significantly worse OS. In the group of AJCC-stage, the patients with the high-risk score in the early stage did not have significantly worse OS (Fig. 7G), while in stage III and stage IV, the patients with the high-risk score have significantly worse OS (Fig. 7 H-I). In the group of AJCC-T, there was no significantly different OS between high- and low-risk score in the T0/1/2 group (Fig. 7J), while in the T3/4 group, the patients with the high-risk score have worse OS (Fig. 7K). Then the risk score of each patient in testing set and entire set was calculated via the coefficient and gene expression of six genes. Then the KM survival analysis was conducted in the entire set, testing set, and external dataset GSE13507 (Fig. 8 A, B, C). Time-dependent ROC analysis was performed to evaluate risk score in the entire set, testing set, and external dataset GSE13507 (Fig. 8 C, D, E). In summary, the six-gene prognostic signature is a reasonably adequate OS predictor for BC patients.

### Construction and validation of the gene-based nomogram

**Fig. 9:**
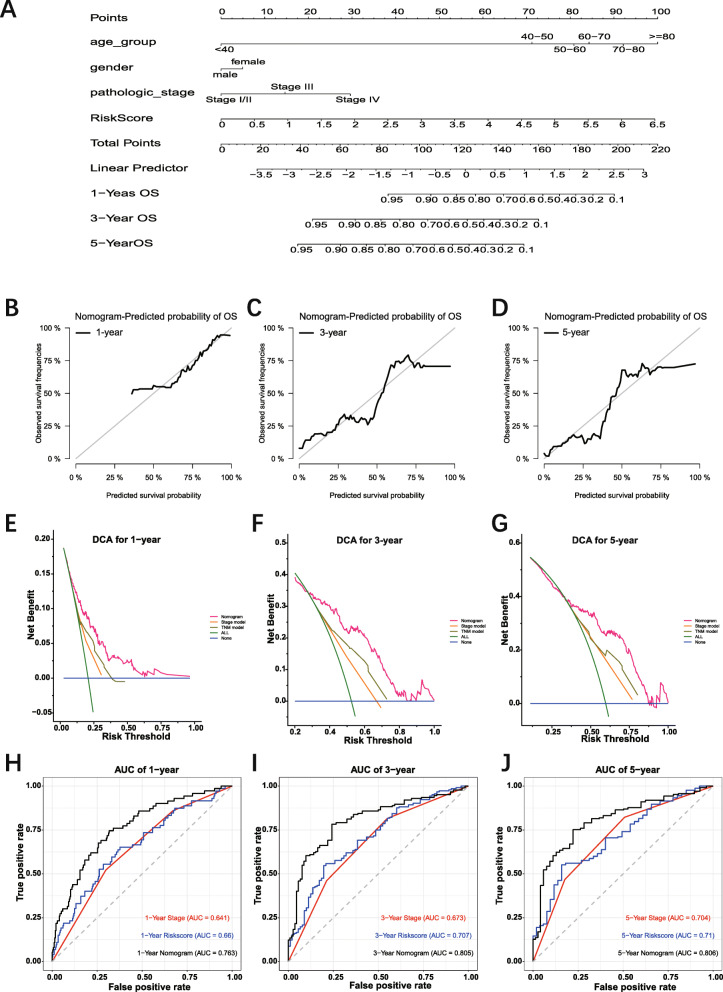
Construction of gene-based prognostic model and evaluation of the nomogram. **A:** Nomogram integrated six-gene based risk score, AJCC pathological stage, gender, and age. **B-D:** The calibration plot of the nomogram for agreement test between 1-, 3- and 5-year OS prediction and actual outcome in TCGA dataset. **E-G:** Decision curve analysis for 1-, 3- and 5-year OS prediction based on nomogram in TCGA dataset. Blue line: assume no patient is at high-risk. Green line: assume all patients are at high-risk. These two lines serve as a reference. Red line: nomogram can provide more net benefits for BLCA patients’ survival prediction. **H:** The time-dependent ROC curves of the nomogram in TCGA dataset. TCGA: the Cancer Genome Atlas. ROC: Receiver operating characteristic

The six-gene prognosis-related signature with other clinical parameters, such as age, gender, AJCC pathological stage, was performed to construct a nomogram to predict 1-year, 3-year, 5-year OS of patients with BC (Fig. 9 A). Considering the accuracy of Cox proportional hazard model, the age and the pathological stage were set polytomous variables in the construction of nomogram [34]. The calibration plot for patient survival prediction suggested that the predicted outcome of the six-gene prognostic nomogram showed consistency with the actual outcome (Fig. 9 B-D). DCA indicated that utilizing the nomogram gained more benefit than utilizing the stage model and TNM model when the threshold probabilities were set more than 0.25 (Fig. 9 E-G). The time-dependent ROC curves indicated that the nomogram had higher predictive accuracy than the stage model and risk score model. The AUC of nomogram time-dependent ROC was 0.763, 0.805, and 0.806 for 1-year, 3-year, and 5-year, respectively (Fig. 9 H-J).

### Functional analysis of genes correlated with 6 prognosis genes

**Fig. 10:**
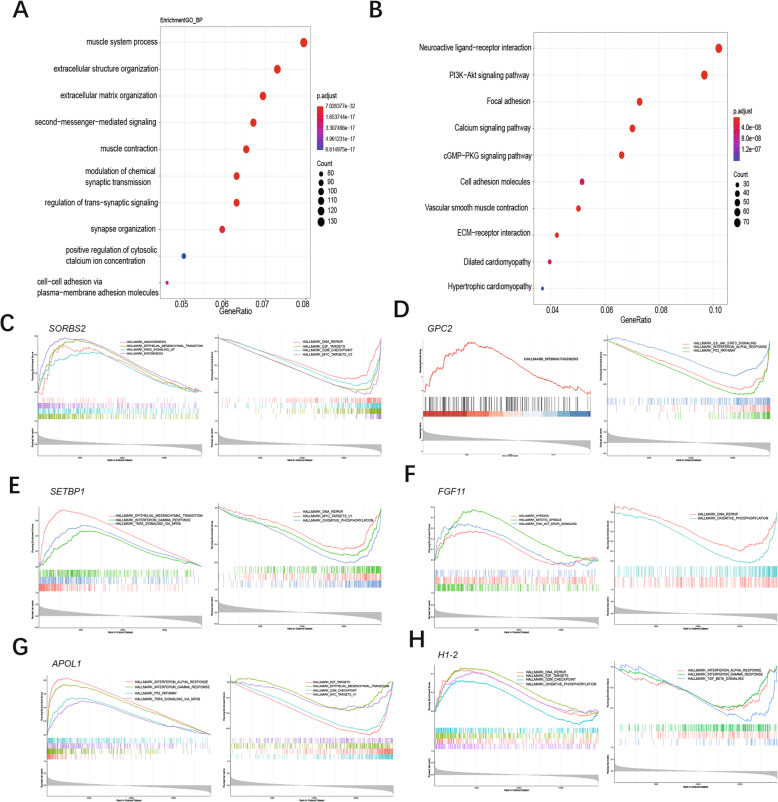
Functional enrichment analysis of genes correlated with prognostic genes. **A:** Top 10 of GO enrichment analysis of these genes. **B:** Top 10 of KEGG enrichment analysis of these genes. **C-H:** GSEA for genes correlated with SORBS2, GPC2, SETBP1, FGF11, APOL1, H1–2. GO: Gene Ontology, KEGG: Kyoto Encyclopedia of Genes and Genomes, GSEA: gene set enrichment analysis

GO and KEGG enrichment analysis was utilized to explore the biological function of genes correlated with candidate genes. In GO biological analysis, these genes were enriched in extracellular structure organization, extracellular matrix organization, second messenger mediated signaling et al. (Fig. 10 A, Additional file: Table S1). In KEGG pathway analysis, PI3K-Akt signaling pathway, Calcium signaling pathway, cGMP-PKG signaling pathway, ECM-receptor interaction, et al. were identified for genes correlated with candidate genes (Fig. 10 B, Additional file: Table S2).

GSEA was performed to identify the potential biological process of 6 prognosis-related genes. Results suggested that the samples with high expression of SORBS2, SETBP1 were enriched in epithelial-mesenchymal transition (Fig. 10 C, E). The samples with low expression of *GPC2, H1–2* were enriched in interferon-alpha response (Fig. 10 D, I). While the samples with high expression of *FGF11* were enriched in PI3K-AKT-MTOR signaling and hypoxia (Fig. 10 F). And the samples with high expression of *APOL1* enriched in P53 pathway, interferon-alpha response (Fig. 10 G).

## Discussion

The incidence of BC is a crucial neoplasm among men, with respective incidence and mortality rates of 9.6 and 3.2 per 100,000 in men: about 4 times those of women globally [1]. It is necessary to screen potential prognostic biomarkers and construct satisfying tools to predict the survival of patients with BC.

In the previous study, numerous prognosis predictions of patients with BC are based on clinical information only [5, 35, 36]. TNM staging system is commonly used to predict the prognosis of bladder cancer. However, as we mentioned above, the single clinical parameter has poor power of prognosis prediction [3]. Therefore, combining other prognostic parameters would be the better way to boost the accuracy of prediction.

In our study, the DEGs between normal tissue and tumor were firstly obtained from three datasets. The intersected genes between DEGs and prognosis-related genes sifted out from the training set were analyzed with LASSO-penalized regression and stepwise multiple Cox regression to screened six candidate genes (*SORBS2, GPC2, SETBP1, FGF11, APOL1, H1–2*). As we are concerned, the method of screening candidate genes via intersecting DEGs and prognosis-related genes was not similar to most bladder cancer prediction model research. The six genes, except *SORBS2*, are significantly related to the overall survival of patients with bladder cancer.

*GPC2*, glypican 2, is a type of cerebroglycan related to oncoprotein. Bosse et al. showed that *GPC2* can be a candidate immunotherapeutic target in High-Risk neuroblastoma [37]. Shou et al. showed that SETBP1 mutation is associated with a poor prognosis in patients with myelodysplastic syndromes [38]. However, the role of *GPC2* and *SETBP1* in urothelial carcinoma is not certain due to the lack of sufficient studies. *FGF11*, fibroblast growth factor 11, is a member of the fibroblast growth factor (FGF) family. Researchers reported that *FGF11* acts as a novel modulator of hypoxia-induced tumor progression [39, 40]. *APOL1*, apolipoprotein L1, encodes a secreted high-density lipoprotein, which binds to apolipoprotein A-I. Some researches indicated *APOL1* is related to cardiovascular disease and renal disease [41, 42]. *H1–2*, H1.2 linker histone, is also called *HIST1H1C*. Li et al. reported that inhibition of H1.2 phosphorylation at T146 was related to the carcinogenic role of K-Ras-ERK1/2 signaling in bladder cancer [43]. This aspect of *H1–2* was also verified in our analysis that the hazard ratio (HR) of H1–2 was significantly less than 1 (Fig. 3 E) and the patients with low *H1–2* expression had a high probability of death, which means the low expression of *H1–2* is related with progression and bad prognosis of patients with BC.

Among these five genes (*GPC2, SETBP1, FGF11, APOL1, H1–2*) related to the prognosis of patients with BC, there are no reports or experiments about these genes related to bladder cancer, except for H1–2. Based on our analysis, these genes may be a potential novel therapeutic target for patients with BC. The mechanism of these four genes is worth to be explored.

The KM survival analysis for the training set and risk stratification in patients with gender, age, race, AJCC stage, AJCC-T, AJCC-N, AJCC-M showed that the risk score had relatively median accurate OS prediction. As for the patients in the T0/1/2 group, low-risk group had worse OS than high-risk group. The reason was that the number of patients with T0/1/2 was probably insufficient, and the bias of this subgroup was enlarged. The time-dependent ROC indicated that the AUC of the nomogram was larger than that of the risk score, resulting from the combination with clinical parameters. It is reasonable that age is an essential risk factor in the progression and prognosis of patients. Some researchers also demonstrated that senescence was associated with a pathological process such as cancer [44]. Therefore, the six-gene-based prognostic nomogram can assist clinicians in predicting the survival outcome of BC patients and provide a more reliable reference for therapy guidance than the single conventional clinical parameter. Besides, these six genes have not been previously studied as prognostic genes in BC patients. To some extent, it is necessary to conduct the following functional experiment exploration based on these six prognostic genes.

The limitations of this study are supposed to be discussed. Although we screened and identified six genes potentially related to the progression and prognosis of patients with BC via some statistical methods and we explored the potential pathways and mechanism of each gene, this study is lacking experiments (in vivo *and* in vitro *validation*) to validate the link between these genes and BC. Therefore, these analyses can be our follow-up studies.

## Conclusion

In our current study, we screened six novel prognosis-related DEGs from the public database and constructed a six-gene-based prognostic nomogram that contained other clinical parameters, such as age, gender, pathological stage, to predict the 1-year, 3-year, 5-year OS of patients with BC. The estimation showed that the nomogram has relatively stable accuracy in the prediction of OS. That is to say, the six genes could be potential biomarkers in BC and, in clinical practice, the related gene-based nomogram could theoretically be utilized to predict the individual survival rate and facilitate the selection of individual treatment options.

## Supplementary Information

**Additional file 1.** Table S1: Gene ontology (GO) enrichment analysis of genes correlated with candidate genes.

**Additional file 2.** Table S2: Kysoto Encyclopedia of Genes and Genomes (KEGG) pathways analysis of genes correlated with candidate genes.

**Additional file 3 **Fig. S1: Immunohistochemistry (IHC) of four genes in urothelial cancer and urinary bladder. APOL1 (A), GPC2(B), and H1–2 (C) were highly expressed in urothelial cancer. SETBP1 (D) was highly expressed in urinary bladder*.*

## Data Availability

All TCGA related data can be obtained from the Cancer Genome Atlas (TCGA, https://portal.gdc.cancer.gov/). The data of GSE13507 and GSE133624 can be obtained from Gene Expression Omnibus (http://www.ncbi.nlm.nih.gov/geo/).

## Abbreviations

BC: bladder cancer
TCGA: The Cancer Genome Atlas
BLCA: Bladder Urothelial Carcinoma
GEO: Gene Expression Omnibus
DEGs: Differentially expressed genes
OCGs: Overlapping candidate genes
ROC: Receiver operating characteristic
AUC: Area under curve
MSKCC: Memorial Sloan Kettering Cancer Center
AJCC: American Joint Committee on Cancer
OS: Overall survival
HR: Hazard ratio
logFC: Log2-based fold change
LASSO: Least Absolute Shrinkage and Selection Operator
GO: Gene Ontology
KEGG: Kyoto Encyclopedia of Genes and Genomes
GSEA: Gene set enrichment analysis
GBA: Guilt by association
*SORBS2*: sorbin and SH3 domain containing 2
*GPC2*: glypican 2
*FGF11*: fibroblast growth factor 11
*SETBP1*: SET binding protein 1
*APOL1*: apolipoprotein L1
*H1–2*: H1.2 linker histone
