# Supplementary figures and images for: Construction of a novel mRNA-signature prediction model for prognosis of bladder cancer based on a statistical analysis
Source: BMC Cancer. 2021 Jul 27;21:858. doi: 10.1186/s12885-021-08611-z (PMC8314557; doi:10.1186/s12885-021-08611-z)

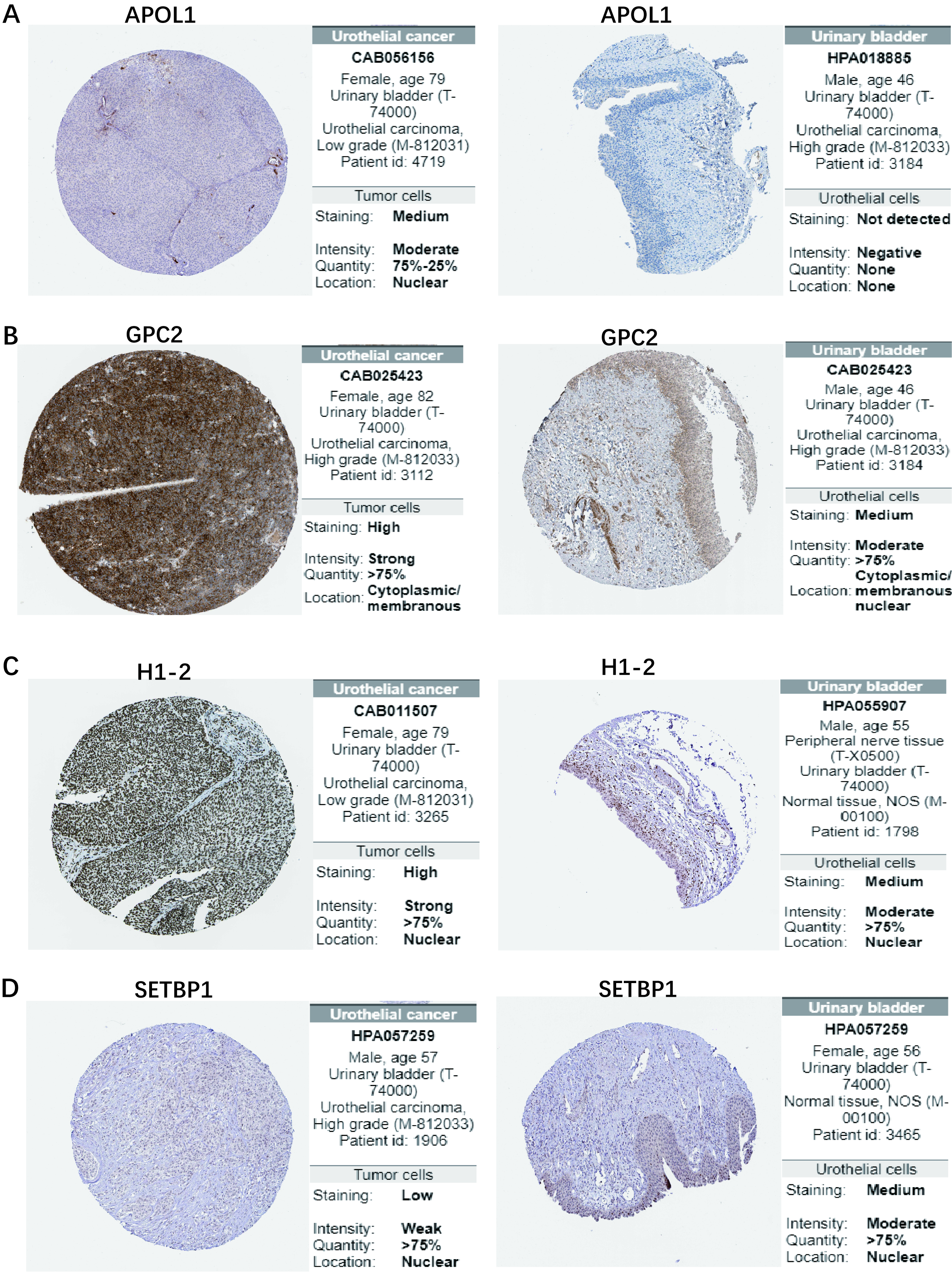

Supplement: Supplementary file 3 — Additional file 3 Fig. S1: Immunohistochemistry (IHC) of four genes in urothelial cancer and urinary bladder. APOL1 (A), GPC2(B), and H1–2 (C) were highly expressed in urothelial cancer. SETBP1 (D) was highly expressed in urinary bladder. [file 12885_2021_8611_MOESM3_ESM.tif]
